# Regional early and progressive loss of brain pericytes but not vascular smooth muscle cells in adult mice with disrupted platelet-derived growth factor receptor-β signaling

**DOI:** 10.1371/journal.pone.0176225

**Published:** 2017-04-25

**Authors:** Angeliki Maria Nikolakopoulou, Zhen Zhao, Axel Montagne, Berislav V. Zlokovic

**Affiliations:** Department of Physiology and Biophysics and Zilkha Neurogenetic Institute, Keck School of Medicine, University of Southern California, Los Angeles, California, United States of America; Hungarian Academy of Sciences, HUNGARY

## Abstract

Pericytes regulate key neurovascular functions of the brain. Studies in pericyte-deficient transgenic mice with aberrant signaling between endothelial-derived platelet-derived growth factor BB (PDGF-BB) and platelet-derived growth factor receptor β (PDGFRβ) in pericytes have contributed to better understanding of the role of pericytes in the brain. Here, we studied *Pdgfrβ*^*F7/F7*^ mice, which carry seven point mutations that disrupt PDGFRβ signaling causing loss of pericytes and vascular smooth muscle cells (VSMCs) in the developing brain. We asked whether these mice have a stable or progressive vascular phenotype after birth, and whether both pericyte and VSMCs populations are affected in the adult brain. We found an early and progressive region-dependent loss of brain pericytes, microvascular reductions and blood-brain barrier (BBB) breakdown, which were more pronounced in the cortex, hippocampus and striatum than in the thalamus, whereas VSMCs population remained unaffected at the time when pericyte loss was already established. For example, compared to age-matched controls, *Pdgfrβ*^*F7/F7*^ mice between 4–6 and 36–48 weeks of age developed a region-dependent loss in pericyte coverage (22–46, 24–44 and 4–31%) and cell numbers (36–49, 34–64 and 11–36%), reduction in capillary length (20–39, 13–46 and 1–30%), and an increase in extravascular fibrinogen-derived deposits (3.4–5.2, 2.8–4.1 and 0–3.6-fold) demonstrating BBB breakdown in the cortex, hippocampus and thalamus, respectively. Capillary reductions and BBB breakdown correlated with loss of pericyte coverage. Our data suggest that *Pdgfrβ*^*F7/F7*^ mice develop an aggressive and rapid vascular phenotype without appreciable early involvement of VSMCs, therefore providing a valuable model to study regional effects of pericyte loss on brain vascular and neuronal functions. This model could be a useful tool for future studies directed at understanding the role of pericytes in the pathogenesis of neurological disorders associated with pericyte loss such as vascular dementia, Alzheimer’s disease, amyotrophic lateral sclerosis, stroke and human immunodeficiency virus-associated neurocognitive disorder.

## Introduction

Pericytes are mural cells of brain capillaries positioned centrally within the neurovascular unit between brain endothelium, astrocytes and neurons [1–4]. They extend their processes along the walls of brain capillaries, and are also found on pre-capillary arterioles, and post-capillary venules [5, 6]. Pericytes receive signals from their neighboring cells and signal back contributing to responses that maintain key neurovascular functions of the brain. This includes angiogenesis and vascular stability during central nervous system (CNS) development [7, 8], formation and maintenance of the blood-brain barrier (BBB) [9–11], and regulation of capillary blood flow [12–15].

Pericyte injury and/or degeneration are found in neurological disorders exhibiting neurovascular dysfunction and BBB breakdown [4]. This includes Alzheimer’s disease [16–21], mild cognitive impairment [22], amyotrophic lateral sclerosis [23, 24], stroke [13, 25, 26], CADASIL (cerebral autosomal dominant arteriopathy with subcortical infarcts), the most common genetic form of ischemic small vessel disease associated with cognitive impairment [27], and human immunodeficiency virus-associated neurocognitive disorder (HAND) [28]. The role of pericyte dysfunction and loss in the pathogenesis of these neurological disorders is, however, still not fully understood.

Studies in pericyte-deficient transgenic mice with aberrant signaling between endothelial-derived platelet-derived growth factor BB (PDGF-BB) and platelet-derived growth factor receptor β (PDGFRβ) in pericytes have contributed to better understanding the role of pericytes in the brain [1, 4]. *Pdgfb* and *Pdgfrβ* homozygous knockout mice completely lack pericytes, which is embryonic lethal causing cerebral blood vessel rupture and microhemorrhages [7, 8]. In contrast, *Pdgfrβ*^*+/-*^ mice and mice with modified PDGF-BB bioavailability are viable, but have reductions in pericyte coverage causing BBB breakdown [9–11]. Additionally, *Pdgfrβ*^*+/-*^ mice develop cerebral blood flow (CBF) dysregulation [15], and over time secondary neurodegenerative changes with a moderate loss of neurons in the cortex and hippocampus [11, 15]. On the other hand, loss of pericytes in mice with diminished PDGF-BB bioavailability leads to different pathology such as calcium deposition in the basal ganglia detectable at 1 year of age [29, 30].

*Pdgfrβ*^*F7/F7*^ transgenic mice carry seven point mutations that disrupt normal PDGFRβ signaling in cells resulting in a partial loss of both pericytes and vascular smooth muscle cells (VSMCs) in the embryonic CNS [8]. These mice also show loss of pericyte coverage in the cortex immediately after birth associated with BBB breakdown [10]. Although loss of pericyte coverage has been reported in the cortex, hippocampus and spinal cord of adult 6–8 month old *Pdgfrβ*^*F7/F7*^ mice [11, 23], little is known as to whether these mice have a stable loss of pericytes in the CNS after birth or alternatively if the brain pericyte population can regenerate and/or compensate for a loss in pericyte coverage. It is also unknown whether VSMCs population is affected in adult *Pdgfrβ*^*F7/F7*^ mice, and/or whether these mice develop progressive vascular phenotype in the brain or not. Here, we report that *Pdgfrβ*^*F7/F7*^ mice show an age-dependent, rapid degeneration of brain pericytes and cell loss leading to progressive capillary reductions and BBB breakdown in some regions more than in others, as for example cortex, hippocampus and striatum compared to thalamus. We also show that the VSMCs population of penetrating arterioles remains unaffected at the time when loss of pericytes is already established in all studied brain regions. Based on these findings, we suggest that *Pdgfrβ*^*F7/F7*^ mice develop an aggressive rapid vascular phenotype without appreciable early involvement of VSMCs, therefore providing a valuable model to study effects of pericyte loss on regional vascular and neuronal functions in the brain.

## Materials and methods

### Animals

Platelet-derived growth factor receptor β mutant mice, *Pdgfrβ*^*F7/F7*^, were generated by point mutations that disrupt the following residues and designated signal transduction pathways; residue 578 (Src), residue 715 (Grb2), residues 739 and 750 (PI3K), residue 770 (RasGAP), residue 1008 (SHP-2), by changing the tyrosine to phenylalanine, and residue 1020 (PLCγ), where tyrosine was mutated to isoleucine [8]. It has been previously shown that loss of seven tyrosine residues on the PDGFRβ results in a severe loss of downstream signal transduction [8]. All mice were maintained on a 129S1/SvlmJ background. Because previous studies did not find that gender affects pericyte coverage or BBB breakdown in mice with disrupted PDGFRβ signaling [11, 23, 31], both male and female mice were used in the present study. Mice at 4–6, 12–16, and 36–48 weeks of age and of both sexes were used in the study. The USC Institutional Animal Care and Use Committee approved all procedures using US National Institutes of Health guidelines (IACUC protocol number 11809). All animals were randomized for their genotype information and were included in the study. The operators responsible for experimental procedure and data analysis were blinded and unaware of group allocation throughout the experiments. All procedures were performed under ketamine/xylazine or isofluorane anesthesia to minimize suffering.

### Immunohistochemistry

Mice were anesthetized intraperitoneally with 100 mg/kg ketamine and 10 mg/kg xylazine to a level of anesthesia such that there is no response to pinch toe, and were transcardially perfused with cold phosphate buffer saline (PBS) containing 0.005 M EDTA at 4°C for 7 min at ~ 3 mL/min using a Rainin Dynamax RP-1 peristaltic pump (Cat #7103–052) to wash out the residual blood from cerebral circulation. After perfusion with cold PBS, mice were perfused for 7 min at ~ 3 mL/min with 4% paraformaldehyde (PFA) for tissue fixation. Brains were post-fixed in 4% PFA overnight at 4°C. Brain tissue was then sectioned at a thickness of 30 μm using a vibratome (Leica VT1000S). Sections were blocked with 5% normal donkey serum (Vector Laboratories)/0.1%Triton-X/0.01 M PBS for 1 h and incubated with primary antibodies diluted in blocking solution overnight at 4°C. We used the following primary antibodies: for pericyte coverage—polyclonal goat anti-mouse aminopeptidase N/ANPEP (CD13; R&D systems, AF2335; 1:250) [9, 19, 23]; for fibrinogen and fibrin extravascular deposits -polyclonal rabbit anti-human fibrinogen (Dako, A0080; 1:250), which recognizes both fibrinogen as well as fibrinogen-derived fibrin polymers and cross reacts with mouse fibrinogen and fibrin [11]; and, for labeling smooth muscle cells, monoclonal FITC conjugated α-smooth muscle actin (SMA) (Sigma, clone 1A4, F3777, 1:100). After incubation with primary antibodies, sections were washed in PBS and incubated with fluorophore-conjugated secondary antibodies for detection of CD13 and fibrinogen and fibrin immunoreactivity. The following secondary antibodies were used: Alexa fluor 647-conjugated donkey anti-goat (Invitrogen, A-21447, 1:500) for CD13; and Alexa fluor 568-conjugated donkey anti-rabbit (Invitrogen, A-10042, 1:500) for fibrinogen and fibrin. To visualize brain endothelial vascular profiles sections were incubated with Dylight 488- or Dylight 594-conjugated *Lycopersicon esculentum* lectin (Vector Labs, DL-1174 or DL-1177; 1:200) for 1 h. For double staining of lectin and CD13, Dylight 488-lectin was incubated simultaneously with Alexa fluor 647-conjugated donkey anti-goat secondary antibody for CD13. For double staining of lectin and fibrinogen and fibrin deposits, Dylight 488-lectin was incubated simultaneously with Alexa fluor 568-conjugated donkey secondary anti-rabbit antibody for fibrinogen. For double staining of SMA and lectin, FITC-conjugated SMA was incubated simultaneously with Dylight 594-conjugated lectin for 1 h. All incubations were performed in dark to prevent fading of fluorescence. All images were taken with a RS-G4 Upright Research Confocal Microscope (Caliber I.D.) and analyzed using ImageJ software (US National Institutes of Health). Gain, digital offset, and laser intensity were kept standardized.

### Confocal microscopy analysis

#### Lasers and Band Pass (bp) Filters

We used a 488 nm laser to excite Alexa Fluor and Dylight 488, and the emission was collected through a 500–550 nm bp filter; a 561 nm laser to excite Alexa Fluor 568 and Cy3 the emission was collected through a 580–630 nm bp filter; a 640 nm laser to excite Alexa fluor 647 and the emission was collected through a 660–720 nm bp filter.

#### Quantification of pericyte coverage and numbers

The quantification analysis of pericyte coverage and numbers was restricted to CD13-positive perivascular mural cells that were associated with brain capillaries defined as vessels with ≤ 6 μm in diameter, as previously described [11, 32].

For *pericyte coverage*, ten-micron maximum projection z-stacks (area 640 x 480 μm) were reconstructed, and the areas occupied by CD13-positive (pericyte) and lectin-positive (endothelium) fluorescent signals on vessels ≤ 6 μm were subjected separately to threshold processing and analyzed using ImageJ. First, black and white 8-bit images for CD13 and lectin signals were thresholded separately using Otsu’s thresholding plugin [33] that minimize the intra-class variance of the thresholded black and white pixels (**S1 Fig**, **steps 1 &2**). After thresholding, the integrated signal density for each thresholded image was calculated. In order to express the integrated signal density as the area of the image (in pixels) occupied by the fluorescent signal, the integrated signal density was divided by 255 (the maximum pixel intensity for an 8-bit image). The integrated pixel-based area ratios of CD13 and lectin fluorescent signals were used to determine pericyte coverage as a percentage (%) of CD13-positive surface area covering lectin-positive endothelial capillary surface area per field (**S1 Fig, step 3**), as previously reported [11]. In each animal, 4–6 randomly selected fields in the somatosensory cortex S1 region (S1Cx), the CA1 region of the hippocampus and posterior thalamus were analyzed in 4 non-adjacent sections (~100 μm apart), and averaged per mouse.

For *pericyte numbers*, ten-micron maximum projection z-stacks were reconstructed, and the number of CD13-positive perivascular cell bodies that co-localized with DAPI (4',6-diamidino-2-phenylindole)-positive nuclei on the abluminal side of lectin-positive endothelium on vessels ≤ 6 μm counted using ImageJ Cell Counter plug-in, as we have previously described [11, 21]. In each animal, 4–6 randomly selected fields (640 x 480 μm) in the cortex, hippocampus and thalamus regions as above were analyzed in 4 non-adjacent sections (~100 μm apart), and averaged per mouse. The number of pericytes was expressed per mm^2^ of tissue.

#### Microvascular capillary length

Ten-micron maximum projection z-stacks were reconstructed, and the length of lectin-positive capillary profiles (≤ 6 μm in diameter) was measured using the ImageJ plugin “Neuro J” length analysis tool. In each animal, 4–6 randomly selected fields (640 x 480 μm) in the cortex, hippocampus and thalamus were analyzed from 4 non-adjacent sections (~100 μm apart), and averaged per mouse, as we have previously described [11]. The length was expressed in mm of lectin-positive vascular profiles per mm^3^ of brain tissue.

#### Extravascular fibrinogen and fibrin deposits

For quantification of extravascular fibrinogen and fibrin deposits, we used an antibody that detects both fibrinogen and fibrinogen-derived fibrin polymers (see above). Ten-micron maximum projection z-stacks were reconstructed, and the fibrinogen and fibrin-positive perivascular signals on the abluminal side of lectin-positive endothelial profiles on microvessels ≤ 6 μm in diameter were analyzed using ImageJ [11]. In each animal, 4–6 randomly selected fields in the cortex, hippocampus and thalamus were analyzed in 4 non-adjacent sections (~100 μm apart), and averaged per mouse.

#### Smooth muscle cell wall thickness and number analysis

For analysis of the VSMCs-covered arteriolar vessel wall thickness, we studied penetrating arterioles (> 25 μm in diameter) from the cortical layer 1 in the somatosensory cortex S1 region. We created maximum intensity projection of 30 μm optical stack and 200 μm SMA-positive segments were selected randomly for diameter measurements. For each arteriole we did 4 measurements 50 μm apart. The thickness of the arterial wall was calculated using the following formula:

$$\mathrm{Arteriolar}\;\mathrm{wall}\;\mathrm{thickness}=\frac{\mathrm{Da}-\mathrm{Dv}}{2}(\mathrm{\mu m}),$$

Where Da equals the SMA-positive arteriolar diameter, and Dv equals the lectin-positive endothelial diameter. The surface area of the arteriolar wall was calculated as:

$$\mathrm{Arteriolar}\;\mathrm{wall}\;\mathrm{surface}\;\mathrm{area}=\pi \;x\;Da\;x\;200\;x\;10^{-6}\;({mm}^{2}),$$

Where *π* is a mathematical constant, the ratio of a circle's circumference to its diameter, commonly approximated as 3.14159, and 200 represents length of 200 μm SMA-positive segments used for analysis. The VSMCs number on a given arteriolar segment was then calculated as:

VSMCsnumber=(thenumberofDAPInucleiwithintheSMA-positivecellarea)–(thenumberofDAPInucleiwithinthelectin-positivearea).

The VSMCs numbers were normalized by the surface area of the arteriolar wall and expressed as VSMCs number/mm^2^ of the surface area of the arteriolar wall.

### Laser Doppler flowmetry (LDF)

The cerebral blood flow (CBF) changes after local adenosine applications were determined in anesthetized mice (1% isofluorane) by LDF measurements through an open cranial window, as previously described [15, 34, 35]. Briefly, the tip of the laser-Doppler probe (Transonic Systems Inc., Ithaca, NY) was placed stereotaxically over the center of the open cranial window (center at AP = -0.94 mm, L = 1.5 mm). Adenosine (Sigma) was then superfused at 400 μM concentration over the window and the CBF responses recorded, as described [15, 34]. The percentage increase in CBF after adenosine administration was expressed relative to the baseline CBF values, and determined from the stable maximum plateau CBF increases within 60 s of adenosine application. Three trials were averaged per animal to obtain the individual values per mouse.

### Statistical analysis

Sample sizes were calculated using nQUERY assuming a two-sided alpha-level of 0.05, 80% power, and homogeneous variances for the 2 samples to be compared, with the means and common standard deviation for different parameters predicted from published data and our previous studies. Dallal and Wilkinson approximation to Lilliefors' method [36] was used to test normality of the data (GraphPad Prism). For multiple comparisons, one-way analysis of variance (ANOVA) followed by Bonferroni's post hoc test was used to test statistical significance between control and mutant mice as well as to test for age-related differences within the mutant group using GraphPad Prism software. For comparisons between two groups, F test was conducted to determine the similarity in the variances between the groups that are statistically compared, and statistical significance was analyzed by Student’s t-test. An investigator blinded to the experimental conditions performed all analyses. Data are presented as mean ± SEM as indicated in the figure legends. *P* value < 0.05 was considered statistically significant.

## Results

### Early and progressive loss of brain pericytes and capillary reductions in *Pdgfrβ*^*F7/F7*^ mice

We focused on several brain regions such as cortex and hippocampus that are frequently affected in murine models of Alzheimer’s amyloidosis, tauopathy, stroke or brain trauma [2–4, 13, 25, 26], as well as thalamus that is affected by calcification in mice with reduced PDGF-BB bioavailability and pericyte deficiency [29, 30], which has been related to idiopathic basal ganglia calcification or Fahr’s disease, a rare human monogenic disorder [29]. We also studied striatum, a region often affected in murine models of stroke or a neurodegenerative process. Pericyte degeneration or dysfunction in several of the studied brain regions have been shown in Alzheimer’s disease [16–22], stroke [13, 25, 26], CADASIL [27], HAND [28], or Fahr’s disease [29].

**Fig 1:**
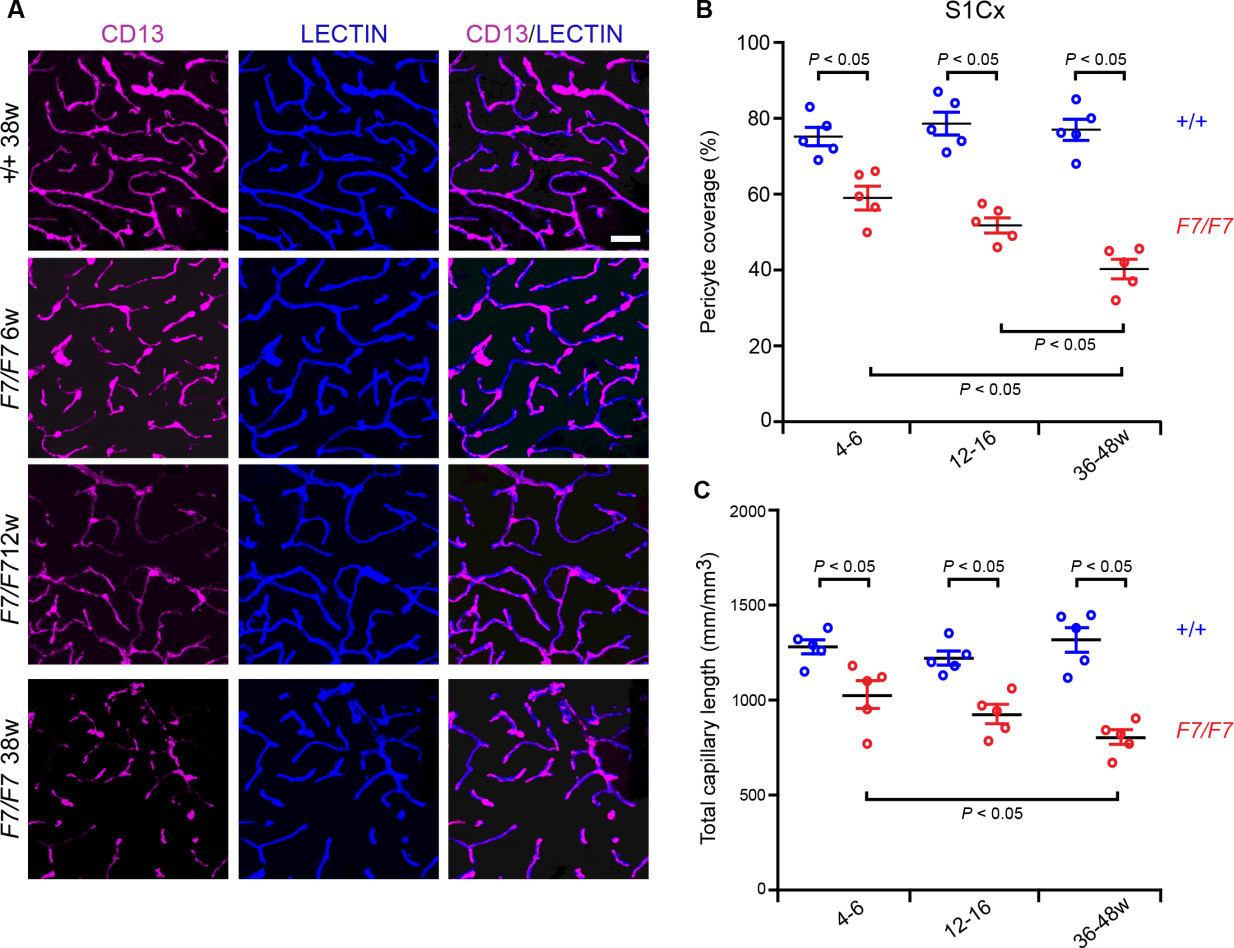
Early and progressive loss of pericyte coverage and capillary reductions in the somatosensory cortex of adult Pdgfrβ^*F7/F7*^ (*F7/F7*) mice. (A) Representative confocal microscopy analysis of coronal sections showing CD13-positive pericyte coverage (magenta, left panels), lectin-positive endothelial vascular profiles (blue, middle panels) and merged (right panels) in the S1 region of the somatosensory cortex (S1Cx) layers II-IV of 6-, 12- and 38-week old F7/F7 and 38-week old control (+/+ 38w) mice. Bar = 40 μm. (B-C) Quantification of pericyte coverage (B) and total capillary length (C) in 4–6, 12–16, and 36-48-week old F7/F7 mice compared to age-matched littermate controls (+/+). Pericyte coverage was determined as a percentage (%) of CD13-positive pericyte surface area covering lectin-positive endothelial surface (See Methods and **S1 Fig**). Total capillary length was determined in mm of lectin-positive endothelial profiles of vessels ≤ 6 μm in diameter, and expressed per mm3 of cortical tissue. In each animal, 4–6 randomly selected fields were analyzed in 4 non-adjacent sections (~100 μm apart), and averaged per mouse to obtain individual values that were taken for statistical comparisons. Mean ± S.E.M., n = 5 animals per group. In B and C, one-way ANOVA and Bonferroni’s post hoc tests were used to compare data in F7/F7 mutants versus age-matched littermate controls and/or between different age groups of F7/F7 mutants only. P < 0.05 indicates statistically significant differences between groups.

Using confocal imaging analysis for CD13, an established pericyte marker [9, 23], along with endothelial-specific *Lycopersicon esculentum* lectin fluorescent staining to visualize brain endothelial profiles [11], we found a substantial loss of pericyte coverage and loss of lectin-positive capillaries (≤ 6 μm in diameter) in *Pdgfrβ*^*F7/F7*^ mice, as illustrated in the somatosensory cortex S1 region (S1Cx) of a 38-week old *Pdgfrβ*^*F7/F7*^ mouse compared to the corresponding age-matched littermate control (**S2A Fig**, low magnification). Representative higher magnification images (**Fig 1A**) and quantification of pericyte coverage (**Fig 1B**) determined as a percentage (%) of CD13-positive pericyte surface area covering lectin-positive endothelial surface area (see **S1 Fig** and Methods for details), confirmed a progressive loss of pericyte coverage with age in the cortex as illustrated in S1Cx region layers II-IV of 4–6, 12–16, and 36-48-week old *Pdgfrβ*^*F7/F7*^ mice compared to the corresponding age-matched littermate controls by 22%, 35% and 46%, respectively. We next found that the total capillary length determined as the length of lectin-positive endothelial profiles of all vessels ≤ 6 μm in diameter, was also progressively reduced with age in the cortex of *Pdgfrβ*^*F7/F7*^ mice, as illustrated by 20%, 25%, and 39% reductions in the S1Cx region layers II-IV of 4–6, 12–16, and 36-48-week old *Pdgfrβ*^*F7/F7*^ mice compared to the age-matched littermate controls, respectively (**Fig 1B and 1C**).

**Fig 2:**
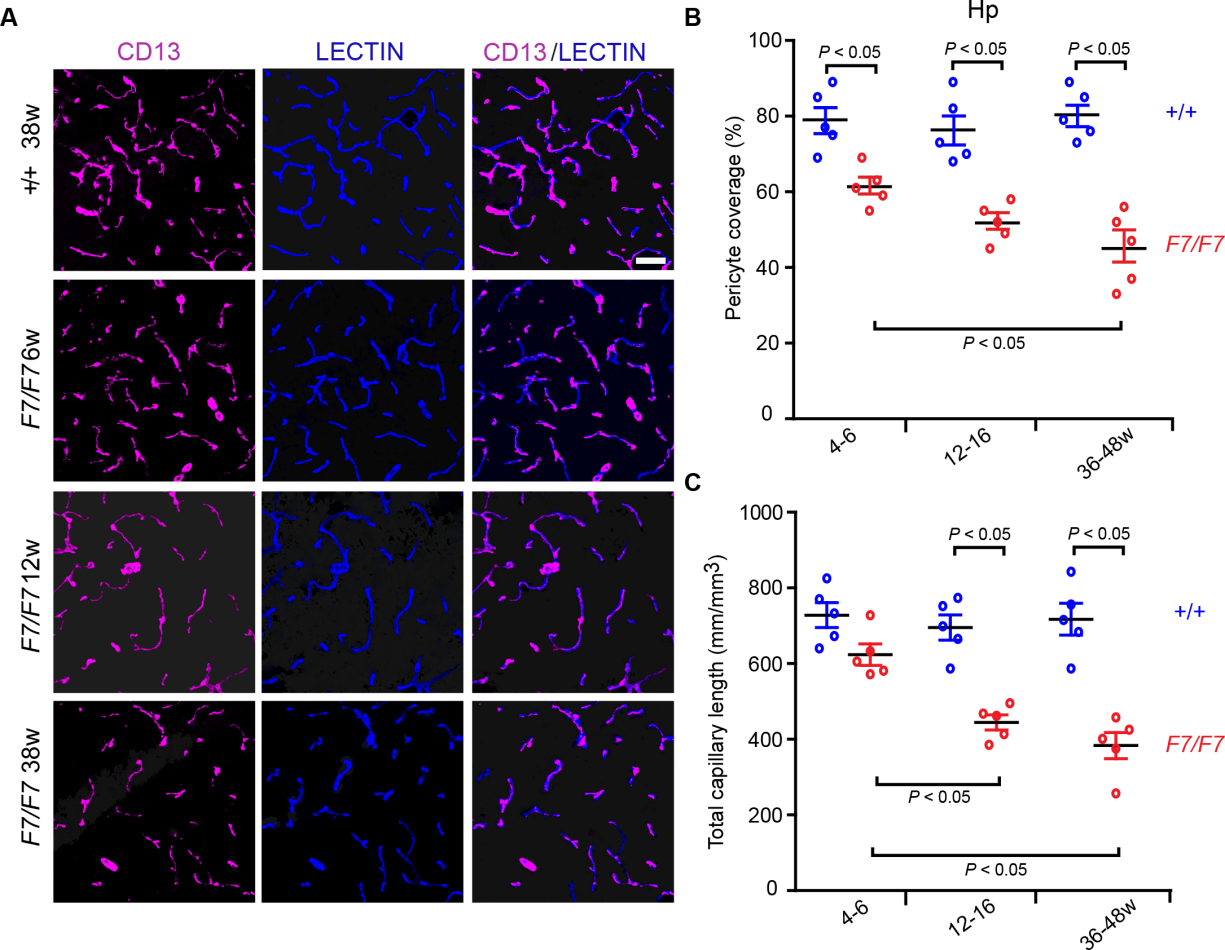
Early and progressive loss of pericyte coverage and capillary reductions in the hippocampus of adult *Pdgfrβ*^*F7/F7*^ (*F7/F7*) mice. (A) Representative confocal microscopy analysis of coronal sections showing CD13-positive pericyte coverage (magenta, left panels), lectin-positive endothelial vascular profiles (blue, middle panels) and merged (right panels) in the CA1 subfield stratum pyramidale of the hippocampus (Hp) of 6-, 12- and 38-week old F7/F7 and 38-week old control mice (+/+ 38w). Bar = 40 μm. (B-C) Quantification of pericyte coverage (B) and total capillary length (C) in 4–6, 12–16, and 36-48-week old F7/F7 mice compared to age-matched littermate controls (+/+) determined as in Fig 1. Mean ± S.E.M., n = 5 animals per group. In B and C, one-way ANOVA and Bonferroni’s post hoc tests were used to compare data in F7/F7 mutants versus age-matched littermate controls, and/or between different age groups of F7/F7 mutants only. P < 0.05 indicates statistically significant differences between groups.

**Fig 3:**
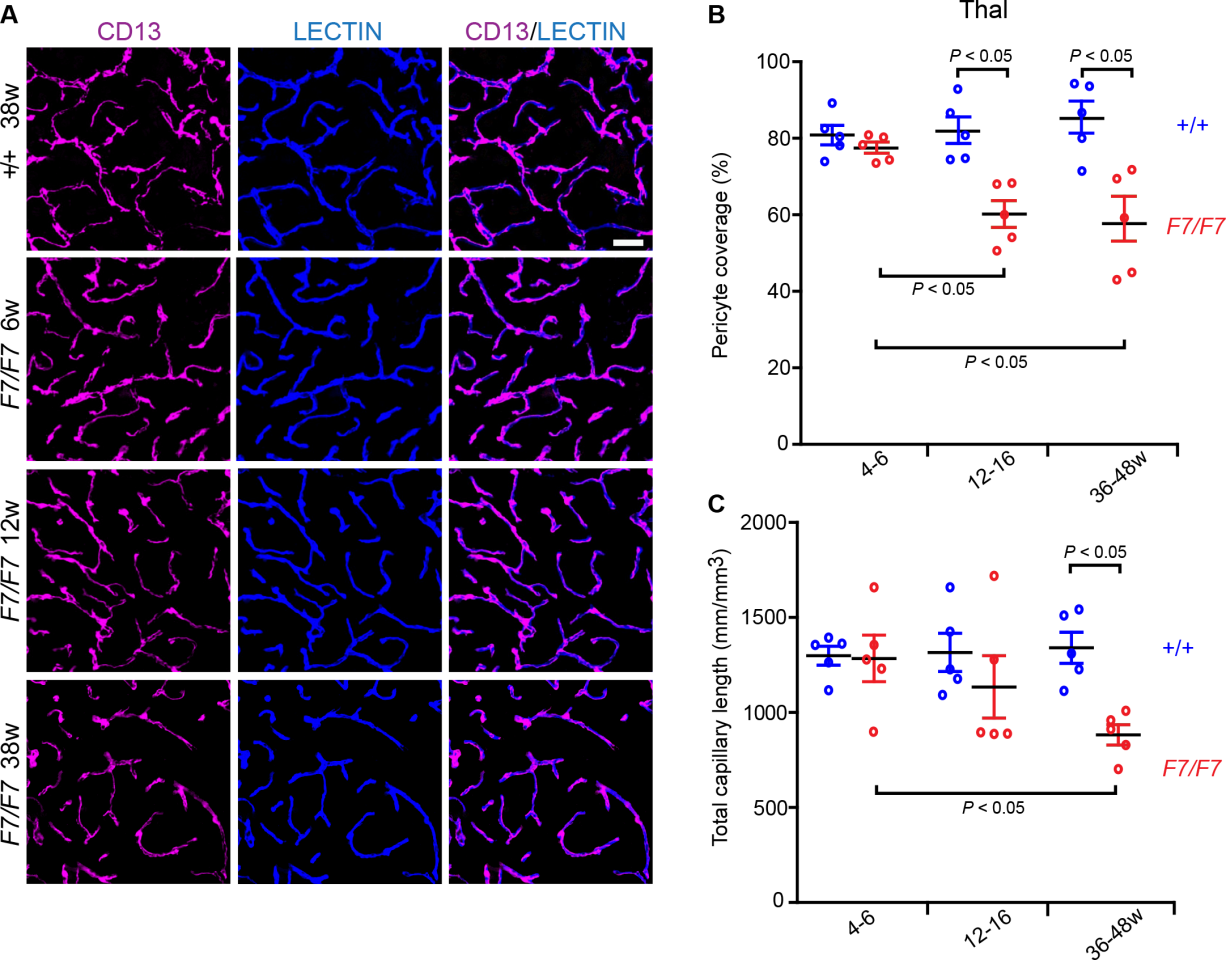
Loss of pericyte coverage and capillary reductions in the posterior thalamus of adult *Pdgfrβ*^*F7/F7*^ (*F7/F7*) mice. (**A**) Representative confocal microscopy analysis of coronal sections showing CD13-positive pericyte coverage (magenta, left panels), lectin-positive endothelial vascular profiles (blue, middle panels) and merged (right panels) in the posterior thalamus of 6-, 12- and 36-week old *F7/F7* and 36-week old control mice (*+/+* 36w). Bar = 40 μm. (**B**-**C**) Quantification of pericyte coverage (**B**) and total capillary length (**C**) in 4–6, 12–16, and 36-48-week old *F7/F7* mice compared to age-matched littermate controls (+/+) determined as in Fig 1. Mean ± S.E.M., n = 5 animals per group. In **B** and **C**, one-way ANOVA and Bonferroni’s post hoc tests were used to compare data in *F7/F7* mutants versus age-matched littermate controls, and/or between different age groups of *F7/F7* mutants only. *P* < 0.05 indicates statistically significant differences between groups.

Progressive reductions in pericyte coverage and total capillary length were also found in the hippocampus of *Pdgfrβ*^*F7/F7*^ mice (**S2B Fig**, low magnification shows the entire hippocampus coronal sections) displaying 24%, 31% and 44% loss of pericyte coverage (**Fig 2A and 2B**), and 13%, 37% and 46% decrease in the total capillary length (**Fig 2A and 2C**) as illustrated in the CA1 region stratum pyramidale in 4–6, 12–16, and 36-48-week old *Pdgfrβ*^*F7/F7*^ mice compared to the corresponding age-matched littermate controls, respectively. Interestingly, loss of pericyte coverage and capillary length in the thalamus developed more slowly than in the cortex and hippocampus, as shown by 4%, 16% and 31% loss in pericyte coverage (**Fig 3A and 3B**), and 1%, 13% and 30% reduction in the total capillary length (**Fig 3A and 3C**) in *Pdgfrβ*^*F7/F7*^ mice compared to the corresponding age-matched controls at 4–6, 12–16, and 36–48 weeks of age, respectively. Loss of pericyte coverage and reductions in microvascular density in the thalamus became, however, more appreciable in 36-48-week old *Pdgfrβ*^*F7/F7*^ mice (**S2C Fig**, low magnification; **Fig 3A, 3B and 3C**). Loss of pericyte coverage and capillary reductions were also found in the striatum (**S3A and S3B Fig)** and were comparable to those reported in the cortex (**Fig 1**) and hippocampus (**Fig 2**).

**Fig 4:**
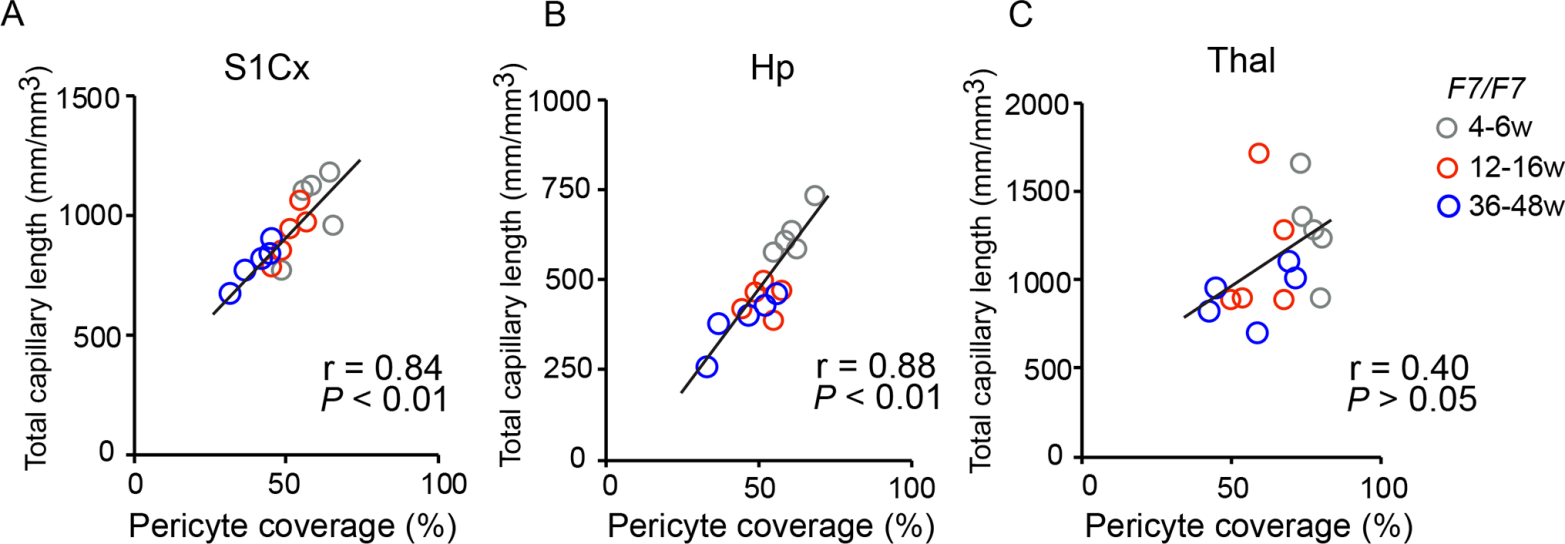
Loss of pericyte coverage correlates with capillary reductions in the cortex and hippocampus of adult *Pdgfrβ*^*F7/F7*^ (*F7/F7*) mice. (**A-C**) Correlations between age-dependent reduction in capillary length and loss of pericyte coverage in the somatosensory cortex S1 region (S1Cx) (**A**), the CA1 region of the hippocampus (Hp) (**B**), and posterior thalamus (Thal) (**C**) in *F7/F7* mice. Single data points were from 4-6- (grey), 12–16 (red), and 36–48 (blue) week-old *F7/F7* mice (n = 15 individual points; each point represents the mean value per mouse calculated as explained in Fig 1 legend and Methods). r = Pearson’s coefficient. *P*, significance.

Reductions in capillary length in *Pdgfrβ*^*F7/F7*^ mice correlated well with the loss of pericyte coverage in the cortex and hippocampus, i.e., the greater the loss of pericyte coverage the greater the loss in the total capillary length (**Fig 4A and 4B**). However, this correlation did not hold in the thalamus over the studied period of time (**Fig 4C**). This could possibly be explained by relatively modest loss of pericyte coverage in the thalamus at early stages compared to other studied brain regions, which did not lead to a significant loss in capillary length until a later stage between 36–48 weeks of age (**Fig 3C**).

**Fig 5:**
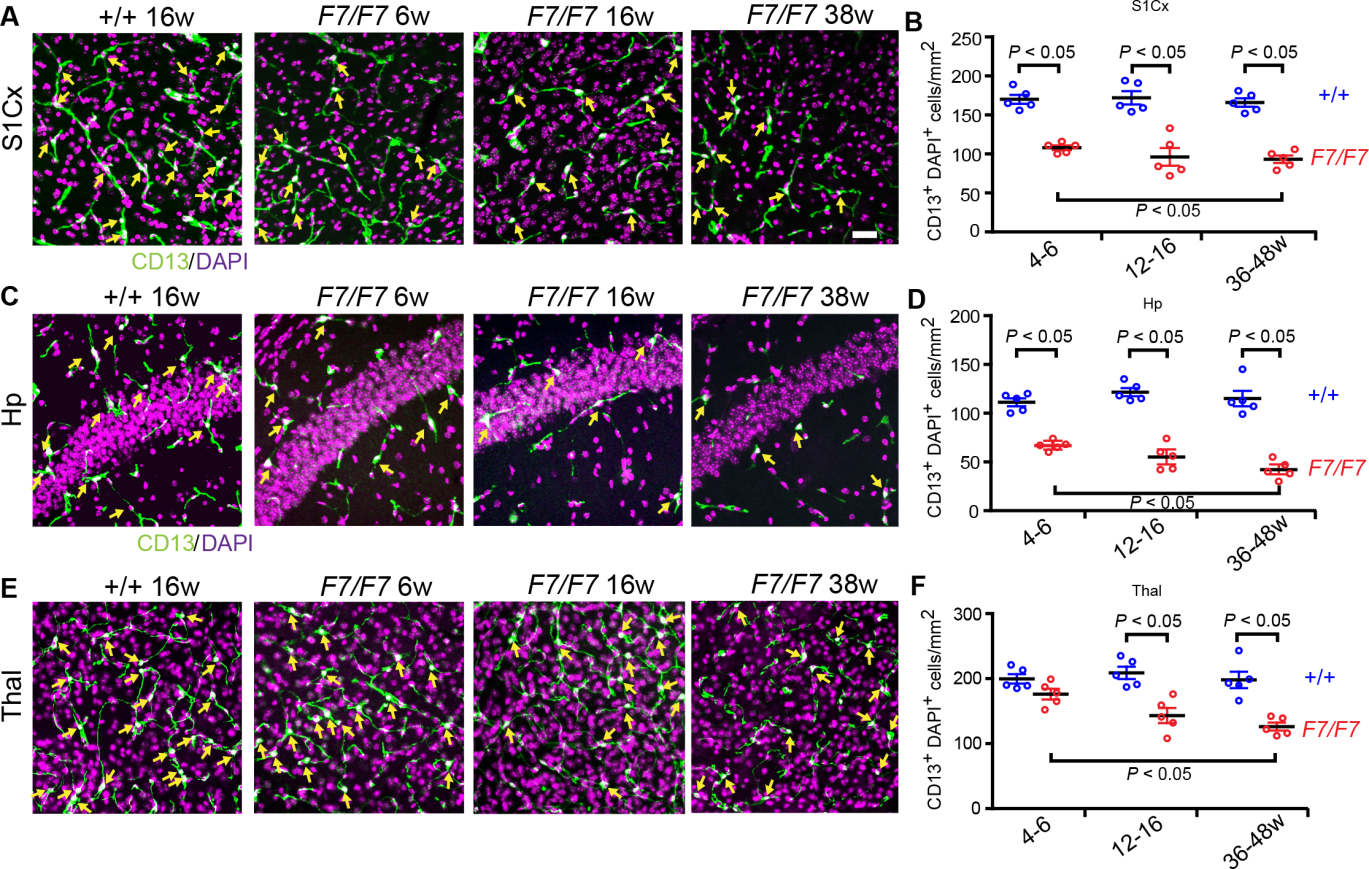
Loss of brain pericytes in adult *Pdgfrβ*^*F7/F7*^ (*F7/F7*) mice. (**A, C, E**) Representative confocal images of CD13-positive pericyte cell bodies co-localized with nuclear 4,6-Diamidino-2-phenylindole, dihydrochloride (DAPI) staining indicated by yellow arrows in the somatosensory cortex S1 region layers II-IV (S1Cx) (**A**), the CA1 subfield of the hippocampus (Hp) (**C),** and posterior thalamus (Thal) **(E)**. Bar = 40 μm. (**B, D, F**) Quantification of CD13-positive cell bodies per mm^2^ of tissue sections in S1Cx (**B**), Hp (**D**) and Thal (**F**). In each animal, 4–6 randomly selected fields in the cortex, hippocampus and thalamus were analyzed in 4 non-adjacent sections (~100 μm apart) and data were averaged per mouse. Mean ± S.E.M., n = 5 animals per group. In **B**, **D**, and **F** one-way ANOVA and Bonferroni’s post hoc tests were used to compare data in mutants versus age-matched littermate controls, and/or between different age groups of *F7/F7* mutants. *P* < 0.05 indicates statistically significant differences between groups.

Consistent with loss of pericyte coverage, pericyte cell numbers determined by counting of CD13-positive pericyte cell bodies that co-localized with nuclear 4,6-Diamidino-2-phenylindole, dihydrochloride (DAPI) staining per mm^2^ of tissue showed 36%, 43% and 49% loss in the cortex (**Fig 5A and 5B**), 34%, 42% and 64% loss in the hippocampus (**Fig 5C and 5D**), and a more moderate and slower 11%, 32% and 36% loss in the thalamus (**Fig 5E and 5F**) of 4–6, 12–16, and 36-48-week old *Pdgfrβ*^*F7/F7*^ mice compared to the age-matched controls, respectively.

### Blood-brain barrier breakdown to blood-derived fibrinogen

**Fig 6:**
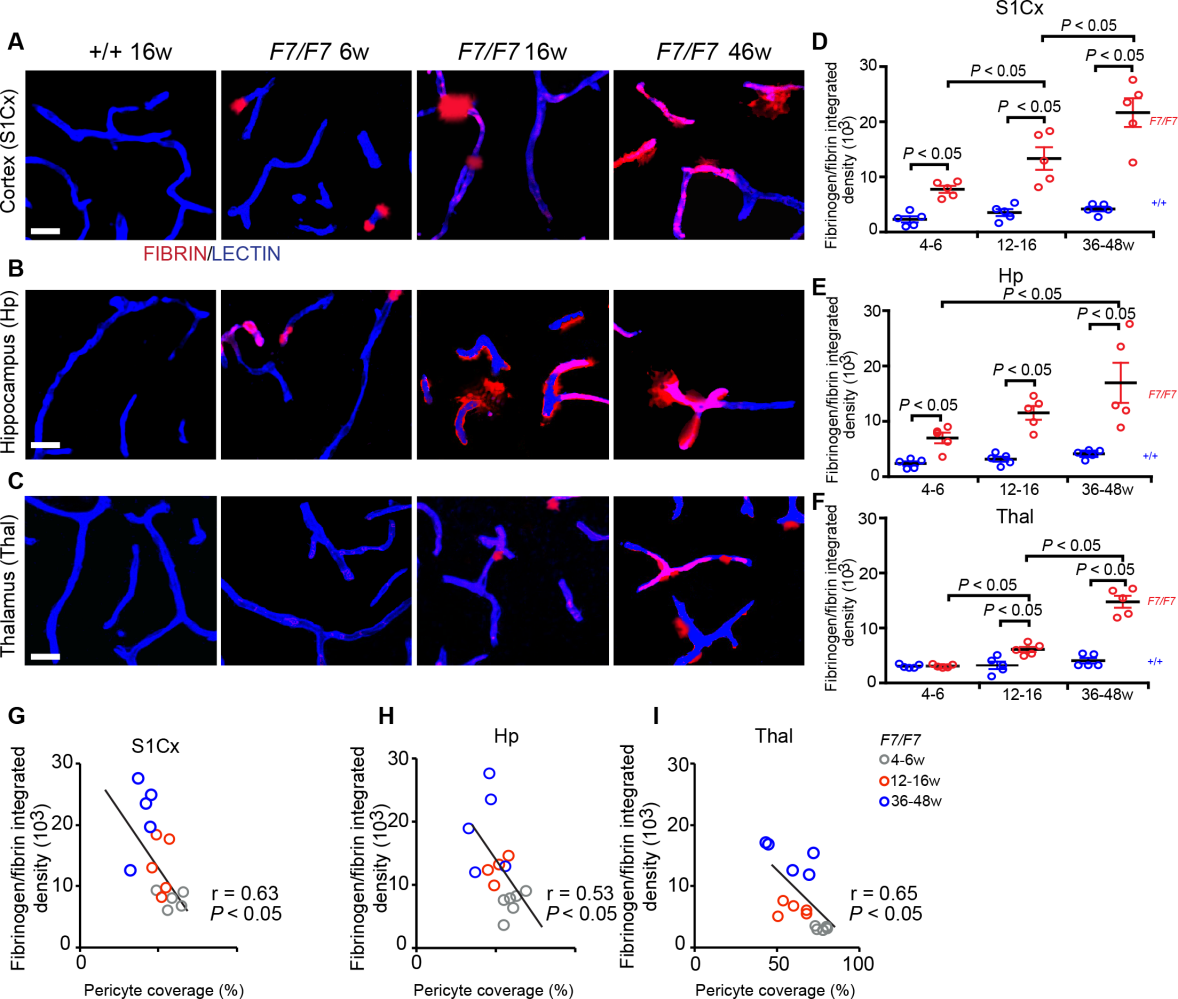
Accumulation of fibrinogen and fibrin perivascular deposits in the brain of adult *Pdgfrβ*^*F7/F7*^ (*F7/F7*) mice. **(A-C)** Representative confocal images of lectin-positive endothelial profiles and extravascular fibrinogen and fibrin deposits in the somatosensory cortex S1 region (S1Cx) layer IV (**A**), the CA1 region stratum pyrmidale of the hippocampus (Hp) (**B**) and posterior thalamus (**C**) of 6-, 16- and 46-week old *F7/F7* mice and 16-week old control (*+/+*) mice. Bar = 20 μm. (**D-F**) Quantification of fibrinogen and fibrin-positive extravascular deposits in the S1Cx (**D**), Hp (**E**) and Thal (**F**) of 4–6, 12–16, and 36-48-week old *F7/F7* mice and age-matched littermate controls (*+/+*). Mean ± SEM, n = 5 mice per group. In each animal, 4–6 randomly selected fields in the cortex, hippocampus and thalamus were analyzed in 4 non-adjacent sections (~100 μm apart) and averaged to calculate individual values per mouse. In **D-F**, one-way ANOVA and Bonferroni’s post hoc tests were used to compare data in mutants versus age-matched littermate controls, and/or between different age groups of *F7/F7* mutants. *P* < 0.05 indicates statistically significant differences between groups. (**G-I**) Correlations between age-dependent fibrinogen and fibrin perivascular accumulation and loss of pericyte coverage in the S1Cx (**G**), Hp (**H**), and Thal (**I**) regions in *F7/F7* mice. Single data points were from 4-6- (grey), 12-16- (red), and 36-48- (blue) week old *F7/F7* mice (n = 15 individual points per mouse; r = Pearson’s coefficient; *P*, significance.

Next, we found an age-dependent, progressive BBB leakage of blood-derived fibrinogen, as illustrated in the cortex, hippocampus and thalamus of 6-, 16- and 46-week old *Pdgfrβ*^*F7/F7*^ mice compared to a 16-week old control (**Fig 6A, 6B and 6C**), as well as in the striatum (**S3C Fig**). Quantification of fibrinogen and fibrin perivascular deposits indicated 3.4-, 3.7- and 5.2-fold increase in the cortex (**Fig 6D**) and 2.8-, 3.5- and 4.1-fold increase in the hippocampus (**Fig 6E**) of 4–6, 12–16, and 36-48-week old *Pdgfrβ*^*F7/F7*^ mice compared to the corresponding age-matched littermate controls, respectively. Similar analysis of fibrinogen and fibrin perivascular deposits in the thalamus indicated a more moderate BBB breakdown with undetectable deposits at 4–6 week of age, a moderate accumulation at 12-16-week of age with 1.9-fold increase compared to the age-matched littermates, and a 3.6-fold increase in 36-48-week old *Pdgfrβ*^*F7/F7*^ mice compared to age-matched littermate controls (**Fig 6F**). The magnitude of fibrinogen and fibrin deposits accumulation in the striatum paralleled findings in the cortex and hippocampus (**S3C Fig**).

Accumulation of perivascular fibrinogen and fibrin deposits in *Pdgfrβ*^*F7/F7*^ mice correlated with reductions in pericyte coverage in all studied brain regions (**Fig 6G, 6H and 6I**), i.e., the greater the loss of pericyte coverage the greater the accumulation of perivascular deposits. Although, there was also a correlation in the thalamus (**Fig 6I**), the accumulation of fibrinogen and fibrin perivascular deposits was, however, less prominent than in the cortex and hippocampus, consistent with a slower pericyte degeneration and loss.

### Normal vascular smooth muscle cell numbers and functional response

**Fig 7:**
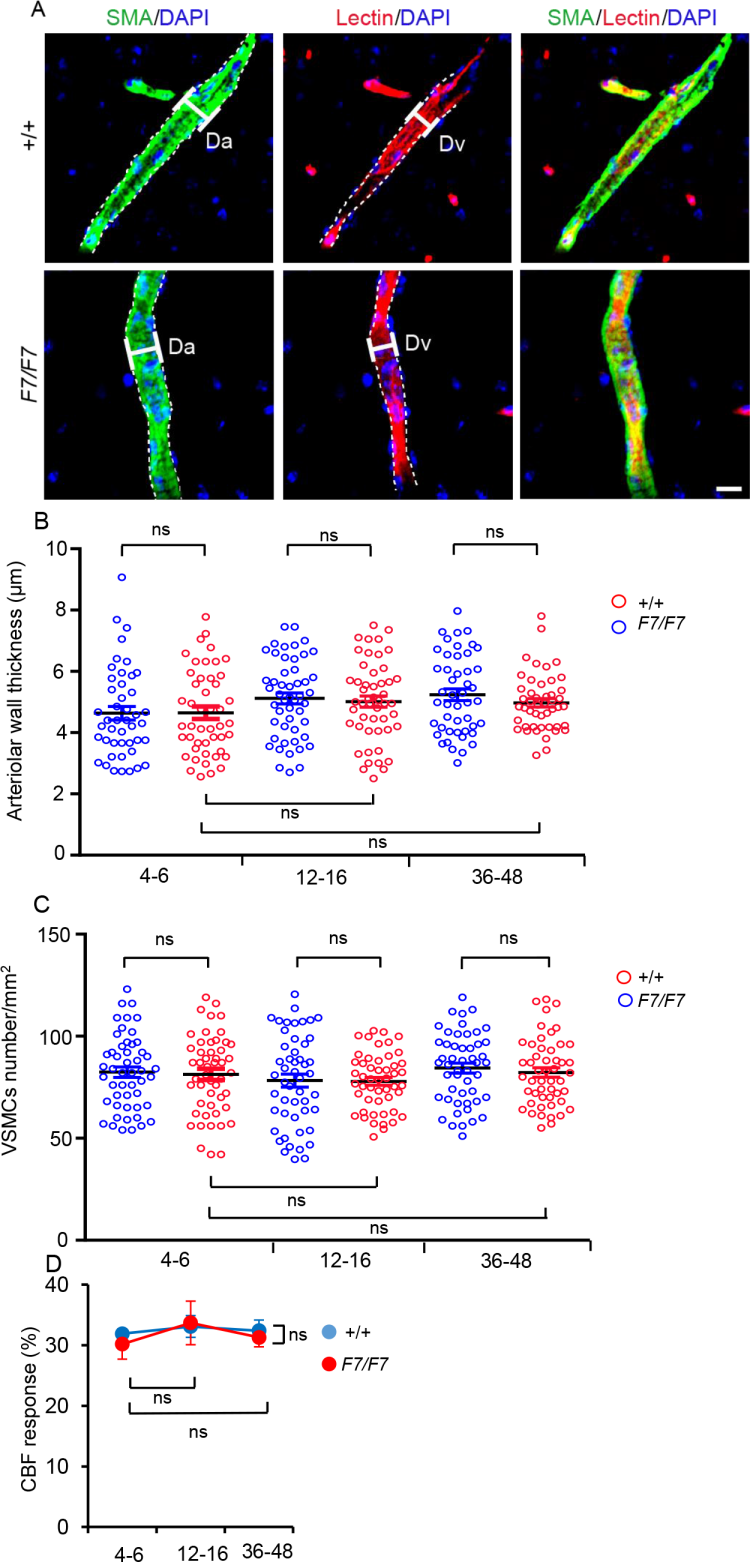
Normal thickness of vascular smooth muscle cells (VSMCs)-covered arteriolar wall, VSMCs cell numbers and functional response to adenosine in adult *Pdgfrβ*^*F7/F7*^ (*F7/F7*) mice. (A) Representative high magnification confocal images (see Methods for details) of α-smooth muscle actin (SMA)-positive VSMCs (green, left panels) and lectin-positive endothelial profiles (red, middle panels), and merged (left panels) in penetrating arterioles of the somatosensory cortex S1 layer 1 cortex (S1Cx) of 12-week old F7/F7 and age-matched littermate control mice (+/+). Interrupted lines indicate the edge of fluorescent signal. Da, SMA-positive arteriolar diameter; Dv, lectin-positive endothelial diameter. Scale bar = 50 μm. (B-C) Quantification of the arteriolar wall thickness (B) and VSMCs numbers (C) in 4-6-, 12-16- and 36-48-week old F7/F7 mice compared to the respective age-matched littermate controls (+/+). In B and C, individual points represent 10 vessels per animal from 5 animals per group. Mean ± S.E.M. from 50 arterioles per group. ns, not significant by one-way ANOVA. See Methods for details. (D) Laser Doppler flowmetry measurements of cerebral blood flow (CBF) response to endothelium-independent VSMCs relaxant adenosine (400 μM) in 4-6-, 12-16-, and 36-48-week old F7/F7 mice compared to age-matched littermate controls (+/+). Mean ± S.E.M.; n = 3 mice per group; ns, not significant by one-way ANOVA.

Finally, we studied whether adult *Pdgfrβ*^*F7/F7*^ mice also develop reduced numbers of VSMCs at the time when they clearly show reductions in pericyte coverage (**Figs 1, 2 and 3**; **S3A and S3B Fig**) and numbers (**Fig 5**) in all studied brain regions. Confocal imaging analysis for α-smooth muscle actin (SMA), an established marker of VSMCs [1, 4], and endothelial lectin in cortical penetrating arterioles in layer 1 of the S1Cx cortex indicated that the thickness of VSMCs-covered arteriolar wall determined as (Da—Dv)/2 (**Fig 7A**—where Da equals the SMA-positive arteriolar diameter, and Dv equals the lectin-positive endothelial diameter, see Methods for details) was not altered in *Pdgfrβ*^*F7/F7*^ mice compared to controls based on measurements in 50 arterioles in *Pdgfrβ*^*F7/F7*^ mutants and 50 arterioles in controls from 5 animals per group (**Fig 7B**), and was in average (mean ± S.E.M.) 4.6 ± 0.2 μm and 4.6 ± 0.2 μm; 5.1 ± 0.2 μm and 5.0 ± 0.2 μm; and 5.2 ± 0.2 μm and 5.0 ± 0.1 μm in 4–6, 12–16, and 36-48-week old *Pdgfrβ*^*F7/F7*^ mice and age-matched littermate controls, respectively.

The number of VSMCs calculated as the number of DAPI nuclei within the SMA-positive cell area minus the number of DAPI nuclei within the lectin-positive area per mm^2^ of the arteriolar wall surface area (see Methods for details) was also not altered in *Pdgfrβ*^*F7/F7*^ mice based on measurements in 50 arterioles in *Pdgfrβ*^*F7/F7*^ mutants and 50 arterioles in controls derived from 5 animals per group (**Fig 7C**), and was in average (mean ± S.E.M), 82 ± 3 and 81 ± 3; 78 ± 3 and 78 ± 2; and 84 ± 3 and 82 ± 2 per mm^2^ of the arteriolar wall surface area in 4–6, 12–16, and 36-48-week old *Pdgfrβ*^*F7/F7*^ mice and age-matched littermate controls, respectively.

Lastly, we performed laser Doppler flowmetry (LDF) measurements in *Pdgfrβ*^*F7/F7*^ mice and age-matched littermate controls to determine CBF response to adenosine (400 μM) that was applied locally by superfusion over an open cranial window, as previously described [15, 34]. The percentage increase (%) in CBF after adenosine administration was similar between the two groups at each studied age (**Fig 7D**) as indicated by comparable CBF increases relative to the baseline (mean ± S.E.M.) of 31.9 ± 0.73% and 30.2 ± 2.49%; 33.1 ± 1.80% and 33.7 ± 3.60%, and 32.4 ± 1.78% and 31.3 ± 1.56% in 4-6-, 12-16-, and 36-48-week old *Pdgfrβ*^*F7/F7*^ mice and the respective age-matched littermate controls, respectively, suggesting normal functional responses of arteriolar VSMCs in *Pdgfrβ*^*F7/F7*^ mutant.

## Discussion

We show that adult *Pdgfrβ*^*F7/F7*^ mice develop a progressive and rapid early vascular phenotype between 4–6 and 36–48 weeks of age with reduction in pericyte coverage and cell numbers, reduction in capillary length (vessels ≤ 6 μm in diameter), and an increase in perivascular fibrinogen and fibrin deposits reflecting BBB breakdown in several studied brain regions including cortex, hippocampus, striatum and thalamus. A similar, but somewhat more moderate loss of pericyte coverage, BBB breakdown and microvascular reductions have been previously reported in pericyte-deficient *Pdgfrβ*^*+/-*^ mice that develop less aggressive vascular phenotype [11, 15]. Microvascular reductions and BBB breakdown have also previously been shown in mice with modified PDGF-BB bioavailability that develop severe loss of pericytes [9].

In the present study, we observed that some brain regions of *Pdgfrβ*^*F7/F7*^ mice develop more rapidly pericyte loss and vascular phenotype than other regions, as for example cortex, hippocampus and striatum compared to thalamus. These findings are consistent with a previous report showing that mice with reduced PDGF-BB bioavailability also have regional differences in pericyte coverage with thalamus showing less severe loss, but more severe deposition of calcium, than the cortex and hippocampus showing more severe pericyte loss, but no deposition of calcium [30]. The reasons for these regional differences in vascular phenotype and brain pathology remain currently unclear, but they likely might reflect regional differences in signaling within the respective regional brain neurovascular units, which should be explored by future studies. At present, the cellular and molecular mechanisms underlying these regional differences remain unclear.

Our data show that in all studied brain regions of *Pdgfrβ*^*F7/F7*^ mice loss in pericyte numbers was somewhat greater than the loss in pericyte coverage in all age groups, which could be eventually attributed to the vascular plasticity of the pericyte pool compensating for a progressive pericyte loss by generating longer pericyte processes. This would be consistent with previous observations in mice with reduced PDGF-BB bioavailability showing greater loss of pericyte cell numbers compared to a loss of pericyte coverage [9].

It has been previously reported that 6–8 month old *Pdgfrβ*^*F7/F7*^ mice (i.e., 36–48 weeks of age) have completely normal hemodynamic, physiological and biochemical parameters, including tests for liver and kidney functions [11]. For example, *Pdgfrβ*^*F7/F7*^ mice compared to age-matched littermate controls do not show differences in the mean arterial blood pressure, systolic and diastolic blood pressure and/or pulse arterial pressure (difference between systolic and diastolic pressure), heart rate, arterial pO2 and pCO2 and pH, respiratory rate, glucose levels in serum and cerebrospinal fluid, and their liver analyses (i.e., alkaline phosphatase, alanine aminotransferase, aspartate aminotransferase, creatine kinase, albumin, total protein, total bilirubin), and kidney analyses (i.e., blood urea nitrogen, creatinine, calcium, and phosphorous) are normal, as reported [11]. Therefore, the presently studied *Pdgfrβ*^*F7/F7*^ model does not have a general systemic perfusion deficit and/or an apparent cardiovascular insufficiency indicating that vascular phenotype that we see in the brain is mainly of local character.

The current models of PDGF-BB and PDGFRβ deficiency have been extremely helpful in understanding the role of pericytes in regulating neurovascular functions of the brain [1, 4, 9–11, 15, 24]. However, these models are not pericyte-specific. For example, PDGFB/PDGFRβ signaling importantly regulates both pericytes and VSMCs during the development of the vascular system in the embryonic CNS [7, 8]. It is conceivable, however, that pericytes and VSMCs in the brains of PDGF-BB/PDGFRβ deficient models are differentially regulated after birth, which would be consistent with their more specialized and differentiated roles in the adult brain compared to their roles in the developing CNS [4, 11]. For example, classical work has shown that in the embryonic CNS pericyte loss in *Pdgfb* and *Pdgfrβ* null mice leads to endothelial cell hyperplasia, suggesting that pericytes control endothelial cell numbers and microvessel architecture, but do not determine capillary density, length or branching [7, 37, 38]. In the adult brain, however, diminished PDGF-BB and partially deficient PDGFRβ signaling in mice lead to reductions in microvascular density, as others [9] and us [11] have reported, and confirmed in the present study in the *Pdgfrβ*^*F7/F7*^ model.

Importantly, the current study found that VSMCs coverage of arteriolar vessels is not altered in adult *Pdgfrβ*^*F7/F7*^ animals, as shown by no change in the thickness of VSMCs layer and no change in the number of VSMCs in the penetrating cortical arterioles in *Pdgfrβ*^*F7/F7*^ mice compared to the age-matched littermate controls at the time when reductions in pericyte coverage and numbers were already established in all studied brain regions. We have also shown that functional responses of VSMCs-covered arterioles are preserved in *Pdgfrβ*^*F7/F7*^ mice as indicated by comparable increases in CBF to adenosine, an endothelium-independent vasodilator that acts as a direct VSMCs relaxant [39]. Altogether, these data suggest that adult *Pdgfrβ*^*F7/F7*^ mice develop early and progressive reductions in pericytes, whereas VSMCs population remains unaffected and these early stages. It has been recently reported that young *Pdgfrβ*^*+/-*^ mice that develop a significant 27% reduction in pericyte coverage compared to age-matched controls, also have normal thickness of VSMCs-covered arteriolar wall and normal functional responses of arterioles to vasoconstrictors or vasodilators [15]. Whether at a later stage the VSMCs pool in *Pdgfrβ*^*F7/F7*^ and *Pdgfrβ*^*+/-*^ mice remains still unaffected or begins showing degenerative changes resulting from either a progressive vascular phenotype driven by pericyte degeneration and/or due to a primary, slow degenerative process caused by PDGFRβ deficient signaling in VSMCs, remains to be determined by future studies.

Future studies should also explore whether *Pdgfrβ*^*F7/F7*^ mice develop aberrant hemodynamic responses to neuronal stimuli, as recently reported in *Pdgfrβ*^*+/-*^ mice [15], which in turn may contribute to neurodegenerative changes and behavioral hippocampal deficits seen in these mice at 6–8 months of age [11]. It would be also interesting to know what are the exact relative contributions of BBB breakdown versus CBF dysregulation to secondary neurodegeneration processes in *Pdgfrβ*^*F7/F7*^ mice, and whether these mice develop region-specific functional and structural brain changes earlier than *Pdgfrβ*^*+/-*^ mice exhibiting less aggressive vascular phenotype.

Some studies have suggested that neuronal progenitor cells (NPCs) in the subventricular zone also express PDGRFβ [40], but studies by others in the embryonic CNS [7], adult CNS [11, 31] or in acutely isolated murine neurons [41] did not confirm these findings. Relevant to this study, however, is also that NPCs do not normally reside in the presently studied brain regions including cortex, hippocampus or thalamus. Nevertheless, future studies should examine the role of PDGFRβ deficiency on NPCs in the subventricular zone of *Pdgfrβ*^*F7/F7*^ mice, and determine whether deficient PDGFRβ signaling influences CNS repair after injury, as for example ischemic stroke that leads to both, a rapid pericyte cell death and dysfunction [13, 25, 26] and migration of NPCs from subventricular zone to the sites of ischemic injury [42].

In summary, our data suggest that *Pdgfrβ*^*F7/F7*^ model is suitable for studying the effects of regional, early pericyte loss and the associated vascular phenotype on brain function and structure. At present, it is unclear whether loss of pericytes in this model is driven by a loss of a trophic support of PDGF-BB/PDGFRβ signaling, or alternatively, by accumulation of blood-derived toxic deposits around microvessels, such as for example fibrinogen, which accelerates microvascular damage [43] and might lead to pericyte cell death. It would be also interesting to find out whether deficient PDGRFβ signaling in pericytes can affect pericyte pool in the white matter of *Pdgfrβ*^*F7/F7*^ mice, and if so, how these changes relate to previously reported neurodegenerative changes described at later stages in these mice between 6–8 months of age [11, 15]. Therefore, *Pdgfrβ*^*F7/F7*^ model should provide a useful tool for future studies directed at understanding the role of pericyte dysfunction and loss in the pathogenesis of neurological disorders associated with pericyte degeneration and/or injury including small vessel disease associated with cognitive impairment and dementia, Alzheimer’s disease, amyotrophic lateral sclerosis, CADASIL, HAND and stroke.

## Supporting information

S1 Fig Steps explaining calculations of pericyte coverage as a percentage of CD13-positive cell surface area occupying lectin-positive endothelial capillary surface area on vessels < 6 μm in diameter using ImageJ software. (PDF)

S2 Fig Age-dependent loss of pericyte coverage and brain capillary reductions in adult *Pdgfrβ^F7/F7^* (*F7/F7*) mice. (**A-C**) Representative low magnification confocal microscopy images of coronal sections showing CD13-positive pericyte coverage (magenta, upper panels), lectin-positive endothelial vascular profiles (white, middle panels) and merged (lower panels) in the entire S1 region of the somatosensory cortex (S1Cx) (**A**, Bar = 150 μm), hippocampus (**B**, Bar = 320 μm) and posterior thalamus (**C**, Bar = 100 μm) of a 38-week old *F7/F7* mouse (*F7/F7* 38w) compared to an age-matched littermate control (*+/+* 38w).
(PDF)

S3 Fig Age-dependent loss of pericyte coverage, capillary reductions and accumulation of fibrinogen and fibrin perivascular deposits in the striatum of adult Pdgfrβ^*F7/F7*^ (*F7/F7*) mice. (A-B) Quantification of pericyte coverage (A) and total capillary length (B) in 4–6, 12–16, and 36-48-week old F7/F7 mice compared to age-matched littermate controls (+/+). Pericyte coverage was determined as a percentage (%) of CD13-positive pericyte surface area covering lectin-positive endothelial surface. Total capillary length was determined in mm of lectin-positive endothelial profiles of vessels ≤ 6 μm in diameter, and expressed per mm3 of cortical tissue. (C) Quantification of fibrinogen and fibrin-positive extravascular deposits in the striatum of 4–6, 12–16, and 36-48-week old *F7/F7* mice and age-matched littermate controls (*+/+*). In each animal, 4–6 randomly selected fields were analyzed in 4 non-adjacent sections (~100 μm apart), and averaged per mouse to obtain individual values that were taken for statistical comparisons. Mean ± S.E.M., n = 5 animals per group. One-way ANOVA and Bonferroni’s post hoc tests were used to compare data in *F7/F7* mutants versus age-matched littermate controls and/or between different age groups of *F7/F7* mutants only. P < 0.05 indicates statistically significant differences between groups.
(TIF)
