# Supplementary material for: Regional early and progressive loss of brain pericytes but not vascular smooth muscle cells in adult mice with disrupted platelet-derived growth factor receptor-β signaling
Source: PLoS One. 2017 Apr 25;12(4):e0176225. doi: 10.1371/journal.pone.0176225 (PMC5404855; doi:10.1371/journal.pone.0176225)
Supplement: S1 Fig — (PDF) [file pone.0176225.s001.pdf]

S1 Fig

Step 1. Images are open in Image J. Example is taken from a control mouse. Bar = 40 μm.

CD13

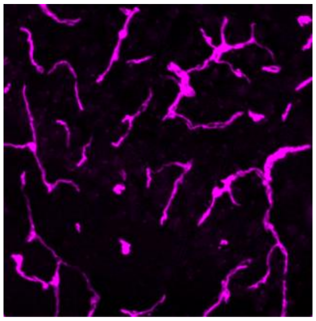

Lectin

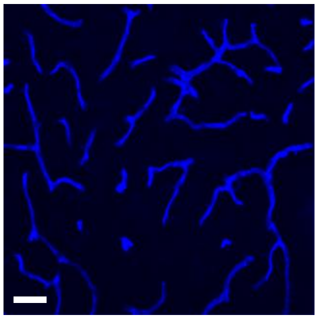

Step 2. Pseudo-colored 8-bit images for CD13 and lectin signals were thresholded using Otsu's thresholding method in ImageJ that minimizes the intra-class variance of the thresholded black and white pixels.

CD13

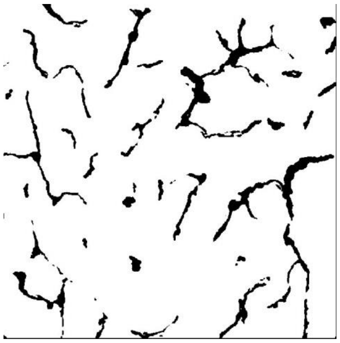

Lectin

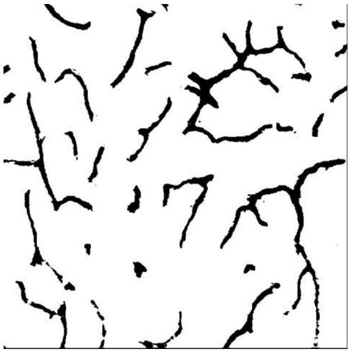

Step 3. The integrated density for each thresholded image was calculated and subsequently converted to a pixel-based area by dividing by 255. For 8-bit images 255 is the maximum pixel intensity. The ratio of CD13-possitive area over lectin-positive area was then calculated as shown in Table below.

	Raw Integrated Signal Density	Area covered by fluorescent signals (pixel-based)	% CD13 signal /Lectin signal
CD13	7226029	7226029 / 255= 94457	94457 / 116500 = 81%
Lectin	8912302	8912302 /255 = 116500	
