# Supplementary figures and images for: Regional early and progressive loss of brain pericytes but not vascular smooth muscle cells in adult mice with disrupted platelet-derived growth factor receptor-β signaling
Source: PLoS One. 2017 Apr 25;12(4):e0176225. doi: 10.1371/journal.pone.0176225 (PMC5404855; doi:10.1371/journal.pone.0176225)

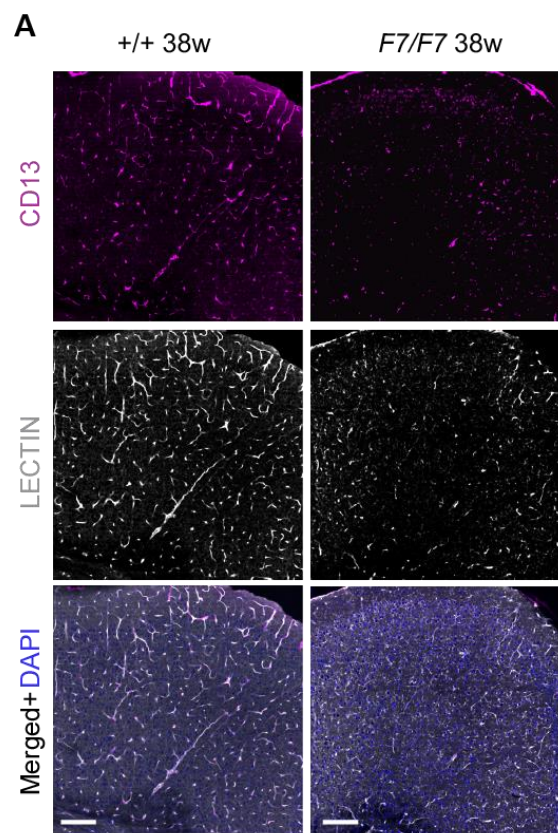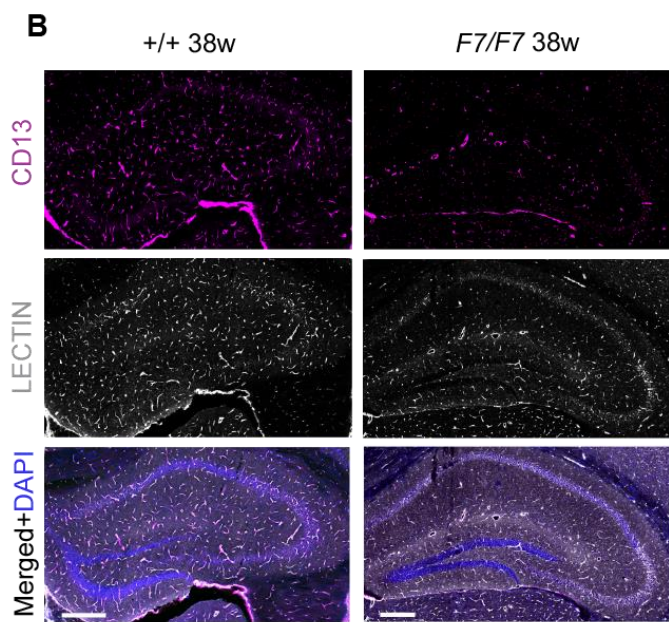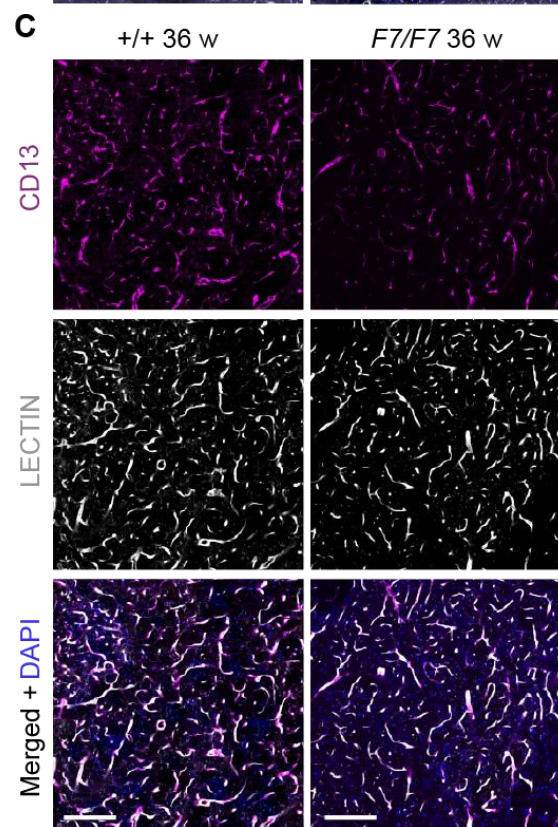

Supplement: S2 Fig — (A-C) Representative low magnification confocal microscopy images of coronal sections showing CD13-positive pericyte coverage (magenta, upper panels), lectin-positive endothelial vascular profiles (white, middle panels) and merged (lower panels) in the entire S1 region of the somatosensory cortex (S1Cx) (A, Bar = 150 μm), hippocampus (B, Bar = 320 μm) and posterior thalamus (C, Bar = 100 μm) of a 38-week old F7/F7 mouse (F7/F7 38w) compared to an age-matched littermate control (+/+ 38w). (PDF) [file pone.0176225.s002.pdf]

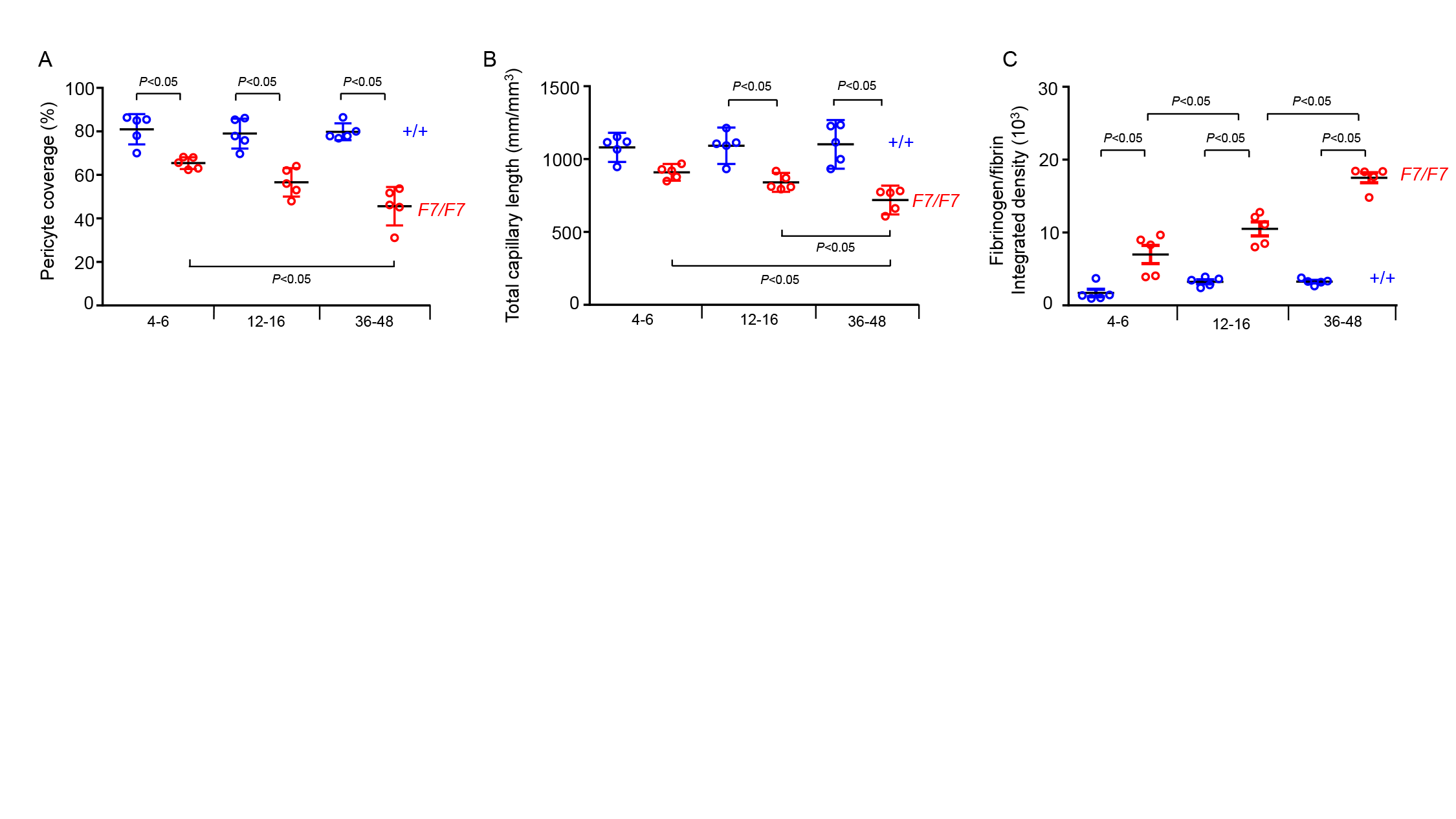

Supplement: S3 Fig — (A-B) Quantification of pericyte coverage (A) and total capillary length (B) in 4–6, 12–16, and 36-48-week old F7/F7 mice compared to age-matched littermate controls (+/+). Pericyte coverage was determined as a percentage (%) of CD13-positive pericyte surface area covering lectin-positive endothelial surface. Total capillary length was determined in mm of lectin-positive endothelial profiles of vessels ≤ 6 μm in diameter, and expressed per mm3 of cortical tissue. (C) Quantification of fibrinogen and fibrin-positive extravascular deposits in the striatum of 4–6, 12–16, and 36-48-week old F7/F7 mice and age-matched littermate controls (+/+). In each animal, 4–6 randomly selected fields were analyzed in 4 non-adjacent sections (~100 μm apart), and averaged per mouse to obtain individual values that were taken for statistical comparisons. Mean ± S.E.M., n = 5 animals per group. One-way ANOVA and Bonferroni’s post hoc tests were used to compare data in F7/F7 mutants versus age-matched littermate controls and/or between different age groups of F7/F7 mutants only. P < 0.05 indicates statistically significant differences between groups. (TIF) [file pone.0176225.s003.tif]
